# Traumatic stress alters neural reactivity to visual stimulation

**DOI:** 10.21203/rs.3.rs-5627085/v1

**Published:** 2025-02-10

**Authors:** Nathaniel Harnett, Grace Rowland, E Kate Webb, Tianyi Li, Soumyaa Joshi, Kerry Ressler, Isabelle Rosso

**Affiliations:** McLean Hospital; McLean Hospital; McLean Hospital; McLean Hospital; McLean Hospital; McLean Hospital; McLean Hospital and Harvard Medical School

**Keywords:** PTSD, Visual stimulation, Cortex, MRI, Trauma

## Abstract

Traumatic stress is a precursor to the development of posttraumatic stress disorder (PTSD). Emergent research suggests visual processing regions may be relevant to PTSD development; however, no previous research to date has investigated the potential effects of trauma exposure on neural reactivity to non-affective visual stimulation. In the present study, 24 recently trauma-exposed (TE) and 16 without recent exposure to trauma (NTE) individuals completed functional magnetic resonance imaging during alternating blocks of flickering checkerboard presentations and rest with an attentional check. TE participants were recruited within 2–4 weeks of trauma, and PTSD symptoms were assessed both at the time of the magnetic resonance imaging scan and 6 months following trauma exposure. TE participants showed greater deactivation within the visual cortex compared to NTE participants. Further, NTE participants showed greater neural reactivity within the dorsomedial prefrontal cortex during stimulation compared to rest, while no difference was observed in TE participants. Connectivity analyses also revealed that visual cortex to paracentral gyrus connectivity was greater during stimulation compared to rest, but only for the NTE participants. Finally, neural reactivity to visual stimulation was negatively associated with PTSD symptoms within the TE group. Our findings suggest that trauma exposure is associated with acute alterations in the neural function that underlies basic visual processing. Furthermore, trauma-induced variability in visual circuit function may be related to the development and expression of PTSD symptoms.

## Introduction

Traumatic stress can result in cognitive and affective dysfunction in the form of posttraumatic stress disorder (PTSD). PTSD is typically thought to be associated with disrupted neurobiological function within core circuitry that supports threat-related processes such as the prefrontal cortex (PFC), hippocampus, and amygdala [1, 2]. However, emergent research suggests that sensory circuits – such as those that support visual processes – are also relevant to the disorder [3, 4]. Limited research to date, however, has probed the neural function of visual circuitry in the early aftermath of trauma to determine if responsivity within such circuitry is either associated with acute traumatic stress or predictive of PTSD symptom development. Further characterization of potential visual circuit dysfunction in recent trauma survivors may thus provide novel insight into the neurobiological mechanisms of PTSD susceptibility.

Prior chronic PTSD research suggests that visual processing of stimuli may be disrupted and associated with neurobiological alterations within visual pathways. For example, previous work observed that visual stimuli (affective and neutral images) were associated with lower neural reactivity in those with PTSD compared to trauma-exposed controls within the visual cortex [5]. Conversely, separate studies observed higher neural reactivity to visual stimuli in those with chronic PTSD [6, 7]. Further, PTSD is associated with altered coactivation patterns within both visual and threat processing networks. A recent report found that coactivation of the visual cortex and hippocampus in individuals with PTSD was associated with intrusion symptoms [8]. Similarly, connectivity between visual networks and prefrontal networks (e.g., frontoparietal network) is predictive of PTSD symptom cluster scores [9]. Contrary findings in the current literature may be partially related to the types of stimulation used to assess affect-related circuitry and responses. Taken together, the prior literature suggests PTSD is associated with altered neural reactivity within both visual and threat processing networks in response to visual stimuli.

Limited work has investigated neural reactivity to visual stimuli in recent trauma survivors. Findings from structural imaging studies suggest the transition from acute stress disorder to PTSD is partially mediated by reductions in gray matter volume of primary visual cortex [10]. Our prior work suggests that structural covariance of the ventral visual stream in recent trauma survivors is associated with PTSD symptoms [11, 12]. Similarly, we have observed that functional coupling between the visual cortex and amygdala/hippocampus in the first few weeks after trauma exposure is associated with 3-month PTSD symptoms when accounting for baseline symptoms [13]. Traumatic stress may therefore be associated with visual circuit neurobiology and development of PTSD symptoms. However, no research has specifically investigated neural reactivity to visual stimulation in recent trauma survivors and whether such reactivity may be related to PTSD symptom development. Further, prior work often examined reactivity to affective stimuli (e.g., negative or fearful images) compared with non-affective stimuli (e.g., neutral pictures or shapes), given PTSD symptoms are frequently tied to negative emotionality. However, it is also possible that the effects of traumatic stress and PTSD are not valence-specific, which may inform neurobiological conceptualizations of PTSD. No research to date has explicitly utilized robust non-affective visual stimuli (e.g., flickering checkerboard) traditionally used to assess visual cortex function to measure potential impacts of recent trauma exposure on reactivity to neutral stimuli. Assessment of neural reactivity to basic, non-affective stimulation may therefore offer novel insight into the neurobiological mechanisms of PTSD susceptibility.

The present study investigated neural responsivity to visual stimulation as a function of recent trauma exposure and PTSD symptom severity. We measured the blood oxygen level dependent (BOLD) response with functional magnetic resonance imaging (fMRI) during a flickering checkerboard paradigm in recent trauma survivors and individuals without recent trauma exposure. We also assessed participants’ self-reported PTSD symptoms at the time of the MRI session and 6 months later. We anticipated that trauma survivors would show altered BOLD responses in the visual cortex compared to control participants and that BOLD responses would correlate with PTSD symptoms. The current findings offer new insight into the acute effects of traumatic stress on the neurobiology of sensorial circuitry and the brain basis of PTSD susceptibility.

## Methods and Materials

### Participants

Both recently trauma-exposed (TE) participants and those without recent exposure to trauma (NTE) participants were recruited for the present study. TE participants were recruited from Emergency Departments (ED) within the Mass General Brigham hospital network via online research invitation or direct contact in participating EDs. Trauma was defined as a medical injury requiring admission to the ED. Details of broad class traumatic events experienced by participants are provided in the supplement (Table S1). NTE participants were recruited via community posting. TE participants were eligible provided they were admitted to a Mass General Brigham facility within the past 2–4 weeks for a traumatic event. NTE participants were included regardless of prior trauma history, provided the trauma had not occurred in the past 2–4 weeks. General inclusion criteria were: a) participant aged 18–65, b) ability to provide informed consent, c) ability to read and speak English, d) an ED Glasgow Coma Scale score of 15, and e) normal or corrected-to-normal vision. Exclusion criteria were: prior intracranial bleeding or hemorrhage, current neurological disorder, mania, psychosis, current suicidal ideation, inability to abstain from drug or alcohol use for four hours prior to study visit, history of schizophrenia or schizoaffective disorder, blood disorder, or an MRI contraindication. Fifty participants were enrolled and consented for the study. Ten participants did not complete MRI and were not included in the present analysis. The final sample consisted of 40 participants (24 TE, 16 NTE; Table 1). All participants gave written informed consent as approved by the Mass General Brigham Institutional Review Board.

### Demographic and psychometric assessment

Participant demographics were assessed using a self-report form that indexed characteristics such as sex assigned at birth, race, ethnicity, age, income, and education. Participant race and ethnicity were assessed via self-report along several categories, with multiple selection allowed. Demographic data are reported in Table 1.

Participants reported lifetime trauma exposure using the Life Events Checklist for DSM-5 (LEC-5)[14] and the Childhood Trauma Questionnaire Short Form (CTQ-SF) [15]. The LEC-5 queries participants on exposure to potentially traumatic events that were a) directly experienced, b) witnessed by the participants, c) learned about as happening to someone close to the participant, or d) encountered through occupational exposure to details of the event due to occupation. Responses on the LEC-5 were summed to obtain an index of prior trauma load for each participant. The short-form version of the CTQ consists of 28 items to assess experiences of abuse (physical/emotional/sexual), neglect (physical/emotional), and minimization/denial. All items were rated on a Likert scale from 1 to 5, and the total score was calculated as the sum of the abuse and neglect subscales (accounting for reverse scored/negatively phrased items). PTSD symptoms were assessed using the PTSD Checklist for DSM-5 (PCL-5) [16]. The PCL-5 is a 20-item self-report questionnaire assessing symptom presence and severity. During the MRI visit, TE participants were asked to answer questions related to the recent event that brought them to the ED, focusing on symptoms since the time of trauma. NTE participants were asked to answer questions related to their worst traumatic event and symptoms over the past month. Both TE and NTE participants completed the PCL-5 again six months later to query symptoms experienced in the past month.

### Magnetic resonance imaging

A T1-weighted multi-echo magnetization prepared rapid acquisition gradient echo (MEMPRAGE) structural scan was acquired (TR = 2500ms, TEs = 1.81/3.60/5.39/7.27ms, TI = 1000ms, flip angle = 8 degrees, FOV = 256mm, slices = 208, Voxel size = 0.8mm isotropic). Functional MRI data was collected using a multi-echo sequence (TR = 2000ms, TEs = 13.00/30.22/47.44/64.66ms, flip angle = 67 degrees, FOV = 208mm, slices = 72, Voxel size = 2.4mm × 2.4mm × 2mm) during visual stimulation (Figure S1). The visual stimulation task consisted of alternating blocks of a flickering checkerboard and a rest/attention check. A full-field flickering checkerboard (8Hz) with a yellow fixation cross was presented for 15 seconds. Immediately following, a red dot on a gray background was presented for 15 seconds. Participants were instructed to fixate on the yellow cross and to press a button on an MRI-compatible button-box when the red dot was presented. Each block was presented 14 times. Preprocessing was completed using FMRIPREP version 21.1.1. Details of the preprocessing steps are provided in the supplement (Supplemental Material). For multi-echo fMRI processing, we calculated a T2* map across echo times to create a single, optimally combined timeseries. The first echo of the fMRI task’s corresponding fieldmap was used for susceptibility distortion correction and applied to the combined timeseries. For the present analyses, non-aggressively denoised outputs from AROMA were used for subsequent first-level models.

Primary first-level level analyses to obtain participant-level maps of blood oxygen level dependent (BOLD) response to stimuli were completed using the Analysis for Functional NeuroImages (AFNI; [17]). Preprocessed data were normalized such that amplitude deflections from the first-level models represented percent signal change. The first-level models included parameters for baseline drift, presentation of the checkerboard condition, presentation of the rest/red-dot condition, participant button presses, as well as global, white matter, and cerebrospinal fluid signal. The checkerboard and rest conditions were modeled by convolving a boxcar function for the duration of the block with a canonical gamma-variate hemodynamic response function (HRF). Button presses were modeled by convolving the instantaneous regressor with the HRF. The other parameters were not convolved with an HRF.

### Statistical analyses

Statistical analyses were conducted using the JASP statistical software package (JASP-stats) and AFNI. Chi-square and independent samples t-tests were performed to evaluate between-group differences in demographic and psychometric assessments between the TE and NTE groups. Voxelwise linear models were run using 3dMVM [18] to evaluate main effects of group (TE versus NTE), stimulus type (stimulation versus rest), and a group by stimulus type interaction on the BOLD response. Residuals from the first-level models were used to determine the spatial autocorrelation function (3dFWHMx) for each participant, and the average was used to define the spatial autocorrelation in 3dClustSim (10,000 iterations) and determine cluster extent (*k* = 172 needed to maintain a = 0.05 (cluster forming threshold of p = 0.005). The BOLD response was extracted for each participant within significant clusters from the voxelwise analysis and included in linear regression analyses to investigate the association between average or differential responses to stimuli with both acute and 6-month PTSD symptoms.

Two *ad hoc* exploratory analyses were performed to determine if neural connectivity may vary as a function of trauma exposure or stimulus type based on our findings from the above model. Using significant regions identified in the 3dMVM above, we first completed an analysis of background connectivity by correlating the timecourse of the residuals from the first-level models within the ROIs identified in the main analyses with the rest of the brain [19]. We also completed a general psychophysiological (gPPI) interaction analysis using ROIs identified in the primary analysis [20]. The gPPI involved detrending and deconvolving (with a standard HRF) the average signal within ROIs across time in the preprocessed fMRI data, combining the resultant neuronal timecourse with dummy-coded interaction regressors for each stimulus presentation, and then reconvolving the waveform with the HRF. The first level models were similar to those above except they also included a general timecourse for the ROI and separate timecourses merged with the dummy-coded contrast files for each stimulus condition (stimulation and rest). We applied the same cluster extent and cluster forming threshold as above (i.e., *k* = 172, p = 0.005).

We next completed linear models to investigate associations between PCL-5 scores and BOLD reactivity in *a priori* ROIs that exhibited significant effects. Average and differential responses were calculated and subject to separate models with the PCL-5 total scores. We investigated associations with PCL-5 subscale scores if significant associations with the total score were observed. A nominal p-value of p = 0.05 was used to determine significance.

## Results

### Behavioral responses to the attention check

All NTE participants had 100% accuracy (i.e., pressed the button during the red dot presentation) during the task (Figure S2). A one-sample t-test within the TE group revealed accuracy was significantly greater than zero [t(23) = 12.06, p < 0.001, M = 84%, SD = 34%] with four participants showing below 90% accuracy. An independent samples t-test on the ratio of button presses to number of trials (assessing multi-button presses during trials) did not reveal a significant difference between the NTE and TE groups [t(38) = 0.06, p = 0.954]. For further fMRI analyses, we set a minimum accuracy cut-off of 50% and excluded four participants in the TE group. For completeness, we also completed sensitivity analyses using the button press ratios as a covariate in our initial voxel-wise linear mixed effects model (see supplementary material).

### Neural responses to visual stimulation

**Figure 1:**
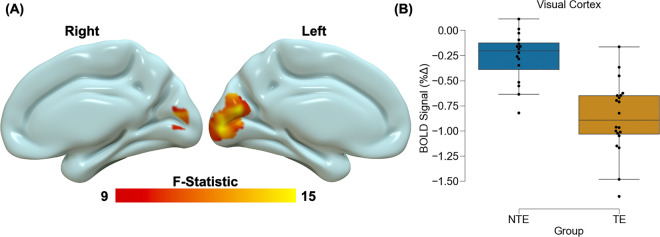
Trauma-related reductions in visual cortex reactivity to stimulation. Recent trauma-exposed (TE) individuals showed lower blood oxygen level dependent (BOLD) signal responses compared to non-recent trauma-exposed (NTE) individuals. The group-effect contrast revealed significant differences between TE and NTE individuals within the visual cortex (A). Resultant signal extraction and descriptive plots of the data revealed the effect was reduced BOLD signal reactivity across all stimuli for the TE group compared to the NTE group (B). Graph depicts the average BOLD signal across the cluster observed in (A). The blue bar represents the NTE group and the orange bar represents the TE group. Black dots represent individual data points for each group. Inner bars within the boxplot represent the mean and outside bars represent the standard error.

**Figure 2:**
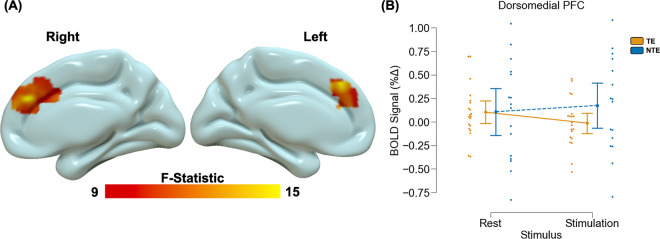
Trauma-related alterations in prefrontal processing during visual stimulation. A voxelwise multivariate model revealed a stimulus by group interaction effect within the dorsomedial PFC (A). Specifically, recent trauma-exposed (TE) individuals showed lower blood oxygen level dependent (BOLD) signal responses to stimulation compared to rest, however non-recent trauma-exposed (NTE) individuals showed greater BOLD signal responses to stimulation compared to rest within this region (B)). Graph depicts the average BOLD signal across the cluster observed in (A). Point plots represent the mean and 95% confidence interval for TE (orange) and NTE (blue) groups during rest and stimulation. Dots represent individual data points for TE (orange) and NTE (blue) during rest and stimulation.

Voxelwise analyses revealed significant main effects of stimulus type across the brain (Table 2). We observed greater deactivation during the rest blocks compared to stimulation blocks within the visual cortex, thalamus/hippocampus, caudate nucleus, and middle occipital gyrus. We also observed greater activation during the rest compared to stimulation blocks within the temporal-parietal-occipital junction, middle temporal gyrus, calcarine gyrus, dorsolateral PFC, insula, cerebellum, lingual gyrus, and superior frontal gyrus. We found a main effect of group within the visual cortex (Fig. 1) such that there was greater deactivation within the visual cortex for TE participants compared to NTE participants. The voxelwise analysis also revealed a significant group by stimulus type interaction within the dorsomedial PFC (Fig. 2). Post-hoc analyses for the interaction effect revealed that TE participants showed greater activation during rest compared to stimulation within the dorsomedial PFC [t(19) = 5.98, p < 0.001_corrected_]. Conversely, NTE participants showed greater activation during stimulation compared to rest [t(15) = −2.96, p = 0.027_corrected_]. However, TE and NTE participants did not show significant between-group differences in dorsomedial PFC responses during rest or stimulation (p > 0.05_corrected_).

### Neural connectivity during visual stimulation

**Figure 3:**
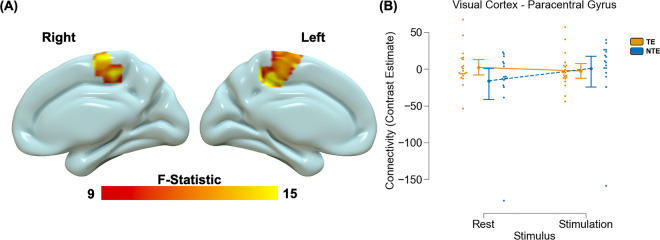
Trauma-related alterations in visual cortex to paracentral gyrus connectivity during visual stimulation. A voxelwise multivariate model revealed a stimulus by group interaction effect on paracentral gyrus to visual cortex connectivity (A). Specifically, recent trauma-exposed (TE) individuals showed no difference in functional connectivity between stimulation compared to rest, however non-recent trauma-exposed (NTE) individuals showed greater connectivity during stimulation compared to rest (B). Graph depicts the average contrast estimate from the general psychophysiological (gPPI) model across the cluster observed in (A). Point plots represent the mean and 95% confidence interval for TE (orange) and NTE (blue) groups during rest and stimulation. Dots represent individual data points for TE (orange) and NTE (blue) during rest and stimulation.

Voxelwise analyses did not reveal significant group differences in background connectivity using either the dmPFC or the visual cortex seed identified in our primary analyses. We did observe a significant main effect of stimulus (Table S2) and a group by stimulus interaction task-related connectivity between the visual cortex ROI and paracentral gyrus (Table S2; Fig. 3). Post hoc analyses revealed TE participants did not show significant task-related differences in coupling between the visual cortex and paracentral gyrus [t(19) = 2.09, p > 0.05_corrected_], while NTE participants showed greater coupling during visual stimulation [t(15) = −6.46, p < 0.001_corrected_]. The pattern persisted even after removing one NTE participant with extreme negative values [t(14) = −6.09, p < 0.001_corrceted_].

### PTSD symptoms and visual stimulation

**Figure 4:**
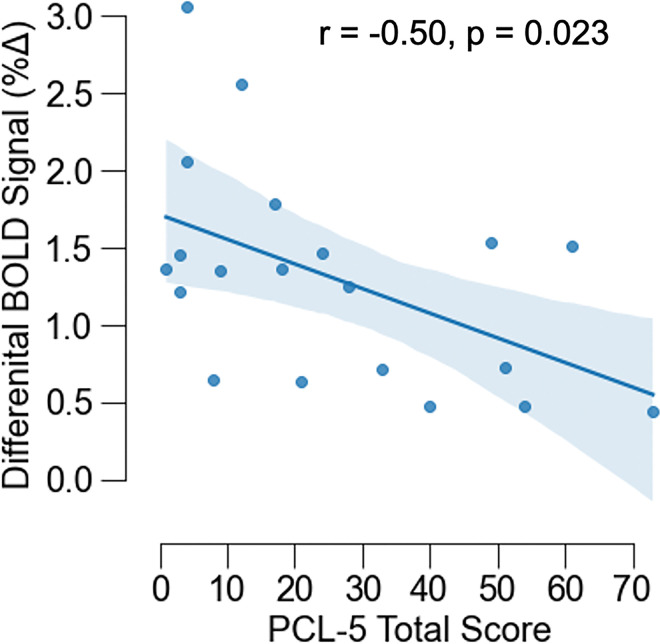
PTSD symptoms vary with differential neural reactivity within the visual cortex. A correlation analysis revealed that, within the TE group, differential (Stimulation – Rest) reactivity within the visual cortex was negatively associated with PTSD symptoms indexed via the PCL-5. Graph depicts the linear association between PCL-5 total scores and differential BOLD signal responses for each participant. Points represent individual data points, the solid line represents the linear line of best fit, and the shaded regions represent the 95% confidence interval.

Given our strong *a priori* hypotheses about visual processing in PTSD, we extracted the BOLD response from the visual cortex identified in the main effect of group and correlated both the differential (checkerboard minus rest) and the average BOLD response with PCL-5 scores within the TE group. PCL-5 scores were negatively correlated with differential BOLD responses (checkerboard minus rest) within the TE group [r(19) = −0.50, p = 0.023] (Fig. 4). Follow-up analyses of symptom subscale scores revealed a significant association of differential BOLD response with intrusion [r(19) = −0.52, p = 0.019], negative cognition and mood [r(19) = −0.50, p = 0.024], and hyperarousal [r(19) = −0.48, p = 0.048] scores, but not avoidance [r(19) = −0.33, p = 0.162] scores. We did not observe a significant interaction between group and the average BOLD response on PCL-5 scores (p > 0.05).

We completed additional analyses with PCL-5 scores collected 6 months after trauma exposure. We did not observe significant associations with either the differential or average BOLD response with the visual cortex ROI (all p > 0.05). We then completed exploratory *ad hoc* analyses using the significant interaction effect on neural reactivity (dmPFC ROI) and connectivity (paracentral gyrus ROI). We observed that 6-month PCL-5 scores were positively correlated with the average connectivity between the visual cortex and paracentral gyrus [r(13) = 0.77, p = 0.001]. The 6-month PCL-5 scores were not correlated with other contrasts (all p > 0.05).

## Discussion

Trauma exposure can lead to debilitating cognitive-affective dysfunction that is likely mediated by alterations in neurocircuitry involved in processing stimuli within the environment. While prior PTSD research has predominately focused on neural processing of affective information within threat neurocircuitry of trauma survivors, limited attention has been paid to processing of non-affective visual stimulation that may activate relevant circuitry for stimulus processing. In the present study, recent TE participants and participants without recent trauma exposure (NTE) completed a visual stimulation task during fMRI. Compared to individuals without recent trauma exposure, recent trauma survivors exhibited greater deactivation within the primary visual cortex and altered neural reactivity to stimulation, compared to rest, within the dorsomedial PFC. Further, connectivity between the visual cortex and paracentral gyrus during stimulation and rest differed as a function of recent trauma exposure. Finally, neural reactivity within visual cortex was associated with PTSD symptoms – particularly intrusion symptoms – within recent trauma survivors. The present findings suggest that trauma exposure may alter processing of basic visual information, potentiating downstream dysfunction in the form of PTSD symptoms.

Recent trauma exposure was associated with greater deactivation in the primary visual cortex. The visual cortex and its connected ventral visual stream form a primary pathway that supports the processing of both neutral and emotionally-valenced objects and other stimuli [21, 22]. There is limited prior work on neural reactivity to non-affective visual stimulation in recent trauma survivors. Prior research found that individuals with PTSD may show disruptions in visual object recognition. Dysfunctional recognition of affective stimuli is a core component of PTSD, with affected individuals often showing threat overgeneralization and impaired extinction recall [23–25]. Recent work also suggests that heightened PTSD symptoms are associated with reduced accuracy in visual object recognition and reduced relative power of evoked responses within prefrontal and temporal cortical areas [26]. Additionally, other research has shown that women with PTSD display increased visual cortex responses during self-referential processing, but decreased responses during evaluative judgments of others [27]. A key difference between the prior PTSD work and the present study is that, here, the trauma-related variability in visual responsivity occurred independently of stimulus valence (i.e., to neural stimulation). Further, in the present sample, PTSD symptoms were negatively related to neural reactivity to non-affective stimulation in recent trauma survivors. One possible interpretation is that recent trauma exposure induces changes in general processing of stimuli, whereas the conversion to PTSD involves a shift towards more specific deficits in processing emotionally significant stimuli.

Recent trauma exposure appeared to moderate both dorsomedial PFC reactivity, and visual cortex to paracentral gyrus connectivity, during stimulation compared to rest. The dorsomedial PFC and its subcomponents are thought to play a role in detection of, and attention to, salient cues in the environment [28]. Further, both PTSD and trauma exposure are associated with disrupted dorsomedial PFC activity [29–31]. In a prior threat conditioning study [29], we observed that TE individuals with lower expectations of experimental threat during presentations of safety showed greater dorsomedial PFC reactivity compared to those who did not. Taken with the present findings, one interpretation is that trauma exposure may induce a transient shift away from cognitive, effortful processing and towards more automatic processing of stimuli. For example, there may be a tendency toward dampened selective attention after trauma exposure, which could impede appropriate responses under task demands. In the present study, recent trauma survivors showed variability in button-press behavior, and individuals without recent trauma exposure showed modulation of visual to paracentral gyrus connectivity during stimulation. The paracentral gyrus includes the motor cortex, which is important for initiating behavioral responses. Thus, trauma exposure may lead to acute impairments in task engagement by disrupting threat-sensory-behavioral circuitry integration. In line with such reasoning, prior experimental work suggests that acute stress, with high task-demands, can lead to shifts towards automatic processing in a prospective memory task [32]. Relatedly, our previously discussed experimental stress exposure has been associated with generalization of prior fear memories [33]. However, a caveat is that our prior work also suggests some TE individuals are able to engage in compensatory neural processes to meet task demands, and the specific mechanisms underlying variability in such behavior remain unclear. Future research is needed to determine the acute effects of trauma exposure on cognitive-affective function.

The present findings should be considered along with several limitations. The sample was composed of individuals recruited from emergency departments, with most individuals experiencing trauma from falls or motor vehicle accidents. Although other studies of trauma have utilized ED-based recruitment for recent trauma survivors [34, 35], our sample does not include other trauma types (e.g., sexual violence) that may impact affective visual circuitry [36, 37]. Additionally, the present design could have been improved by more objective measures of participant performance. While participants were instructed to fixate on a point on the screen, it is possible that TE and NTE participants differed in their eye movements in a manner that affected the results. Using eye-tracking measures alongside visual fMRI tasks in trauma survivors may help establish adherence to the task and provide novel insight into psychobiological impacts of trauma exposure. Finally, our study design was limited to simple visual stimulation to assess neural responsivity. Future research could use more advanced designs to selectively excite specific regions along the ventral visual stream, which may allow for more fine-grained analyses of circuit dysfunction related to trauma exposure.

In conclusion, recent trauma survivors showed differences in neural responsivity to basic visual stimulation compared to individuals without recent trauma exposure. Specifically, we observed altered reactivity within the visual cortex and dorsomedial PFC. Further, visual cortex responsivity in recent trauma survivors was associated with increased PTSD symptom severity. Taken together, our findings suggest that trauma exposure contributes to altered processing and responsivity of basic, non-affective visual information which – in turn – is related to PTSD symptom expression. The present findings provide novel insight into the neural underpinnings of PTSD.

## Figures and Tables

**Table 1:** 

	Trauma-exposed (TE)	Non-recent trauma-exposed (NTE)	χ2/T-statistic, (p-value)
*Demographics*	n = 24	N = 16	
Age	34.94 (14.78)	33.54 (14.69)	0.14, (0.892)
Sex assigned at birth			3.34, (0.068)
*Female*	11 (46%)	12 (75%)	
*Male*	13 (54%)	4 (25%)	
Race			
*White*	13 (54%)	7 (43%)	
*Black*	5 (21%)	3 (19%)	
*Other*	6 (25%)	6 (38%)	
Highest grade level			0.37, (0.830)
*At least high school education (graduate, GED, some college)*	7 (29%)	4 (25%)	
*At least college education (Occupational/Vocational/Associates/Bachelor’s degree)*	10 (42%)	8 (50%)	
*Graduate degree (Master’s, Professional, or Doctoral)*	6 (25%)	3 (19%)	
*Missing/Not Provided*	1 (4%)	1 (6%)	
Income			0.62, (0.539)
*Average*	$119,489	$93,077	
*Median*	$68,000	$50,000	
*Clinical characteristics*			
PCL-5 scores			
2-week	25.73 (22.97)	7.53 (9.13)	2.91, (0.006)
6-month	13.06 (18.28)	6.29 (7.41)	1.30, (0.204)
PROMIS depression			
2-week	17.74 (8.62)	11.87 (5.07)	2.40, (0.021)
6-month	15.41 (7.85)	13.92 (6.98)	0.54, (0.594)
LEC-5 scores	11.52 (4.11)	13.06 (3.13)	2.18, (0.036)
CTQ-SF scores	41.95 (17.03)	33.69 (12.44)	1.65, (0.109)

**Note**: χ^2^ test used for nominal data and independent t-test used for continuous data. CTQ-SF: Childhood Trauma Questionnaire, Short Form; GED: General Education Development; LEC-5: Life Events Checklist for DSM-5; PCL-5: PTSD Checklist for DSM-5; PROMIS: Patient-Reported Outcomes Measurement Information System.

**Table 2:** 

Region (Peak voxel)	Hemisphere	*F*-statistic	Volume (voxels)	Coordinates (MNI)		
Stimulus				X	Y	Z
Primary visual cortex	Bilateral	314.06	15786	−4	−82	−4
SPL*	Right	99.73	6614	20	−58	68
Supramarginal Gyrus	Left	42.4	3884	−58	−38	30
Middle temporal gyrus	Right	40.48	1825	50	−34	−6
Thalamus/Hippocampus*	Right	200.11	1605	24	−28	−2
Precuneus*	Right	32.88	1452	12	−72	30
	Left	99.97	308	−22	−50	12
	Right	49.1	184	26	−46	16
Superior frontal gyrus	Right	45.17	887	28	−10	62
Hippocampus/Parahippocampal gyrus	Left	27.86	397	−16	−22	−18
	Right	38.87	190	16	−22	−20
Paracentral Gyrus	Left	34.23	241	−18	−14	64
Cerebellum	Right	31.05	238	8	−62	−18
Caudate	Right	25.59	200	16	24	14
Middle frontal gyrus	Right	19.92	200	24	54	20
Group						
Primary visual cortex	Bilateral	23.35	245	−4	−76	14
Interaction						
Dorsomedial PFC	Bilateral	26.4	206	4	40	30

Note:

* Indicates cluster extends bilaterally though the peak is located in a specific hemisphere.

F-statistic and coordinates are for the peak voxel in a cluster. Volume is given in *k*, the number of voxels (2×2×2) in the cluster. SPL = Superior Parietal Lobule; PFC = Prefrontal Cortex
